# The Medical Graduates' College and Polyclinic

**Published:** 1909-03-06

**Authors:** 


### The Hospital

A Journal of

The Medical Sciences and Hospital Administration.

New Series. No. 105, Vol. IV. [No. 1177, Vol. XLV.] SATURDAY, MARCH 6, 1909.

### THE MEDICAL GRADUATES' COLLEGE AND POLYCLINIC.

The tenth annual general meeting of the Medical Graduates' College and Polyclinic, which was held on March 1, marks undoubtedly an important point in the history of that institution. Not that the meeting produced any new or striking pronouncement of policy. On the contrary, familiar voices repeated familiar difficulties and expressed familiar aspirations. And a complacent audience showered votes of thanks all round and resolved more or less languidly to hope for the best. Yet in spite of this seeming calm it is beyond question that the Polyclinic has but just passed through a crisis in which its very existence was brought almost to an ignoble end. And it is venturing once more into the open under conditions which may ensure temporary safety but which in themselves are essentially partial and transitory. The question which all friends and well-wishers of the College must ask is, Have the Managing Council defined for themselves a clear and business-like policy which can utilise the present release from financial strain to establish the institution on a reliable and permanent basis? Without such policy it is certain that experience will repeat itself, and financial difficulties will sooner or later bring the Polyclinic to the disaster from which, owing to Sir Jonathan Hutchinson's generosity, it has on the present occasion managed to escape. As, in our view, the institution is one which has already rendered substantial service to the cause of post-graduation education in the metropolis, and as we believe it, if rightly directed, capable of rendering still further service in this direction, we make no excuse for expressing our interest in the shape of a few critical and fundamental propositions. We offer these in no harsh or carping spirit. Our desire, indeed, is to render help in any way possible to us and to see the Polyclinic a financial as well as an educational success.

The first thing, then, we would say, speaking as outsiders, is that one of the conditions of future success is that the Council of Management should clearly appreciate both the scope and limits of the institution placed under their care. The Polyclinic is not a great teaching hospital, and it cannot pretend to conduct the clinical and pathological instruction which such institutions may and do undertake. Hence it is useless, and worse than useless, to suppose that the Polyclinic can satisfy the demands of medical practitioners who have for a time left their practices in the provinces or colonies and come to London to spend some weeks or months in professional studies. For such practitioners there are now admirable arrangements at several of the general hospitals in the metropolitan area, and the opportunities offered by most of the special hospitals are abundant. In all these, clinical cases may be obsedved in sequence and from day to day, new methods of operative and other treatment may be studied, and the teaching of the post-mortem room may be followed. But while the Polyclinic does not meet the wants of the whole-time student, it does most efficiently and satisfactorily meet the wants of those who, engaged in practice or otherwise, have yet an hour or two daily to give to the improvement of their professional knowledge. Its course of afternoon clinics, for example, is unequalled both for variety of experience and excellence of practical teaching and demonstration. In short, in our view, the main function of the Polyclinic is to supply for practitioners residing permanently or for a time in or near London practical opportunities for the study of the diagnosis and treatment of disease. Classes for instruction in clinical pathology and the use of specialised methods of clinical examination may no doubt be added, and possibly the College might be developed as a centre to which many or all of the allied institutions in London might be affiliated. But to regard it as the equivalent of a hospital offering systematic opportunities in its wards and class-rooms is, in our judgment, a profound mistake. And we emphasise this because even yet some of the guides of the Polyclinic proclaim such a view and propose policies based on it. In one of the two directions here discussed the newly elected Council will have shortly to make up its mind. The past has shown some want of steadiness on the point, and the resulting confusion has been only too evident. There is now a chance for the inauguration of a definite and consistent policy.

Next to policy comes finance. In some sense, indeed, finance governs policy, but a harmonious scheme is not possible until policy is defined. Now it is beyond question that in many of its early undertakings the Polyclinic was directed by a sanguine but unpractical spirit. And as a consequence a comparatively large capital debt was accumulated. That debt remains as a burden until this day, and, at least so it seems to us, one of the first conditions of successful management is to secure its disappearance. It was hardly encouraging to note that at the recent annual meeting this subject provoked not a single reference. In saying this, however, we fully recognise that the newly-appointed members of Council have not yet had time or opportunity to appreciate the situation. Before them lies a serious and responsible task. That task demands steadiness of aim and adherence to sound business principles. It is with all goodwill that we here offer our modest homily to the workers in a cause which merits both professional and popular sympathy and support.

